# Sensor-Integrated Inverse Design of Sustainable Food Packaging Materials via Generative Adversarial Networks

**DOI:** 10.3390/s25113320

**Published:** 2025-05-25

**Authors:** Yang Liu, Lanting Guo, Xiaoyu Hu, Mengjie Zhou

**Affiliations:** 1 Department of Computer Science, Worcester Polytechnic Institute, Worcester, MA 01609, USA; harryliu@ieee.org; 2 The Department of Food Science and Human Nutrition, University of Illinois Urbana-Champaign, Champaign, IL 61801, USA; lanting.guo@ieee.org; 3 Department of Chemical Engineering and Materials Science, Stevens Institute of Technology, Hoboken, NJ 07030, USA; xiaoyh5@uci.edu; 4 Department of Computer Science, University of Bristol, Bristol BS8 1QU, UK

**Keywords:** inverse materials design, generative adversarial networks, sustainable food packaging, biodegradable materials, graph neural networks, sensor integration

## Abstract

This study introduces a novel framework for the inverse design of sustainable food packaging materials using generative adversarial networks (GANs) and the recently released OMat24 dataset containing 110 million DFT-calculated inorganic material structures. Our approach transforms traditional material discovery paradigms by enabling end-to-end design from desired performance metrics to material composition. We developed a GAN-driven inverse design architecture specifically optimized for food packaging applications, integrating sensor-derived data on critical constraints such as biodegradability and barrier properties directly into the generative process. This integration occurs at three levels: (1) sensor-measured properties define conditioning targets for the GAN, (2) sensor data train the property prediction network, and (3) sensor-based characterization validates generated materials. An enhanced EquiformerV2 graph neural network was employed to accurately predict the formation energy, stability, and sensor-measurable properties of candidate materials. The model achieved a mean absolute error of 12 meV/atom for formation energy on the OMat24 test set (25% improvement over baseline models), while predictions of sensor-measured functional properties reached $R^{2}$ values of 0.84–0.89 through the integration of experimental measurements and physics-based proxy models. The framework successfully generated over 100 theoretically viable candidate materials, with 20% exhibiting superior barrier properties and controlled degradation characteristics. Our computational approach demonstrated a 20–100× acceleration in screening efficiency compared to traditional DFT calculations while maintaining high accuracy. This work presents a significant advancement in computational materials discovery for sustainable packaging applications, offering a promising pathway to address the urgent global challenges of food waste and plastic pollution.

## 1. Introduction

The global food packaging industry faces unprecedented challenges at the intersection of food security, environmental sustainability, and economic viability [1]. Conventional food packaging materials, predominantly derived from petroleum-based plastics, contribute significantly to environmental pollution, with approximately 40% of all plastic production dedicated to packaging and only 9% of all plastic waste ever produced having been recycled [2]. This alarming reality has intensified research efforts toward developing sustainable alternatives that maintain or enhance the functional properties of traditional packaging while reducing environmental impact [3]. Food packaging materials must fulfill multiple critical functions simultaneously: protecting food from physical damage, extending shelf life by providing barriers against moisture, oxygen, and microorganisms, maintaining food quality while ensuring consumer safety, and increasingly, supporting integration with sensing technologies for quality monitoring [4]. The development of sustainable packaging that satisfies these requirements without compromising environmental sustainability represents a complex materials science challenge [5]. Traditional approaches to materials discovery in this domain have relied heavily on trial-and-error methodologies and incremental improvements of existing materials, resulting in slow progress and limited innovation [6].

Recent advances in computational materials science, particularly the integration of artificial intelligence with materials databases and sensor-derived property measurements, offer promising pathways to accelerate the discovery of novel sustainable packaging materials [7]. The emergence of large-scale materials databases containing millions of structures with density functional theory (DFT) calculations has created unprecedented opportunities for data-driven materials discovery [8]. Notably, Meta’s recent release of the OMat24 dataset, containing 110 million inorganic material structures with corresponding DFT calculations [9], represents a quantum leap in the available data for computational materials discovery. Concurrently, generative models, particularly generative adversarial networks (GANs), have demonstrated remarkable capabilities in generating novel designs across various domains [10]. In materials science, GANs have been employed to generate molecular structures [11], optimize material properties [12], and explore vast chemical spaces efficiently [13]. However, their application to the inverse design of sustainable food packaging materials remains largely unexplored, particularly when coupled with comprehensive materials databases like OMat24.

Given these advances in both materials databases and generative AI, the inverse design paradigm emerges as the logical methodological choice for addressing the sustainable packaging challenge [14,15]. Unlike traditional forward design approaches that necessitate exhaustive screening of millions of candidate materials—a prohibitively time-consuming and computationally expensive process—inverse design begins with desired performance targets and efficiently works backward to identify promising material compositions. This approach is particularly valuable for sustainable food packaging development where contradictory requirements must be simultaneously satisfied: excellent barrier properties during use and controlled degradability after disposal—a challenging multiobjective optimization problem conventional approaches struggle to resolve. Furthermore, by incorporating sensor functionality within the design constraints, the inverse approach enables the creation of truly multifunctional materials that would remain undiscovered through conventional methods. By strategically navigating the vast materials space toward specific performance targets, this methodology dramatically accelerates the materials discovery timeline from decades to months, directly addressing urgent sustainability imperatives in the packaging industry.

In this work, we introduce a novel framework that leverages GANs for the inverse design of sustainable food packaging materials, utilizing the extensive structural and property data from the OMat24 dataset. Our approach fundamentally transforms the traditional forward design paradigm, which progresses from material composition to property prediction, into an inverse design methodology that begins with desired performance characteristics and works backward to identify optimal material compositions [11]. The framework integrates three key innovations: (1) a GAN-driven inverse design architecture specifically optimized for food packaging applications, (2) an enhanced EquiformerV2 graph neural network for accurate prediction of formation energy and stability, and (3) the incorporation of sustainability constraints directly into the generative process. This approach enables the efficient exploration of the vast materials space represented in the OMat24 dataset, with specific focus on identifying materials that exhibit excellent barrier properties while maintaining biodegradability. By bridging the gap between computational materials science and sustainable packaging requirements, our framework addresses the pressing need for rapid development of environmentally friendly food packaging solutions. The integration of GANs with the OMat24 dataset not only accelerates the discovery of novel materials but also provides insights into structure–property relationships that can guide future experimental efforts. Furthermore, our approach demonstrates substantial computational efficiency gains compared to traditional DFT calculations, enabling broader exploration of candidate materials within practical time constraints.

This paper is organized as follows: Section 2 investigated related literature regarding the food packaging and materials discovery. Section 3 introduced the key concepts and techniques employed in our method design, including density function theory, generative adversarial networks, graph neural networks, and sensor technology. Section 4 describes the methodology, including the GAN architecture, the EquiformerV2-based property prediction model, and the incorporation of sustainability constraints. Section 5 presents the results of our computational studies, including the prediction accuracy of our model, the properties of generated candidate materials, and computational efficiency comparisons. Section 6 discusses the implications of our findings for sustainable food packaging development and outlines future research directions. Finally, Section 7 concludes with a summary of our contributions and their potential impact on addressing global challenges related to food packaging sustainability.

## 2. Related Works

### 2.1. Sustainable Food Packaging Materials

Recent years have witnessed substantial research efforts directed toward developing sustainable alternatives to conventional plastic food packaging. Biodegradable polymers derived from renewable resources have emerged as promising candidates, with polylactic acid (PLA), polyhydroxyalkanoates (PHAs), and starch-based materials receiving significant attention [16]. Despite their environmental advantages, these materials often exhibit inferior barrier properties compared to conventional plastics, limiting their practical applications in food packaging [5]. Various strategies have been employed to enhance their functional properties, including the incorporation of nanofillers [3], the development of multilayer structures [17], and surface modifications [18].

Inorganic materials have also been explored for sustainable packaging applications, primarily as barrier-enhancing additives or coatings. Layered silicates, metal oxides, and carbon-based nanomaterials have demonstrated the ability to improve barrier properties when incorporated into biopolymer matrices [19]. However, the systematic exploration of inorganic materials specifically designed for food packaging applications remains limited, particularly for materials that combine excellent barrier properties with controlled degradability [20].

### 2.2. Computational Materials Discovery

The emergence of high-throughput computational methods has revolutionized materials discovery across multiple domains. Materials databases, such as the Materials Project [8] and OQMD [21], have enabled researchers to access DFT-calculated properties for thousands of materials, accelerating the identification of candidates for various applications. More recently, Meta’s release of the OMat24 dataset, containing 110 million inorganic material structures with corresponding DFT calculations, has dramatically expanded the available data for computational materials discovery [9].

Machine learning approaches have been increasingly employed to predict material properties from structural data, enabling rapid screening of candidate materials without the computational expense of DFT calculations. Graph neural networks, in particular, have demonstrated remarkable success in capturing the complex relationships between atomic structure and material properties [22]. The EquiformerV2 architecture, which incorporates equivariant transformers to model geometric relationships in 3D structures, represents a significant advancement in this domain, achieving state-of-the-art performance on various materials property prediction tasks [23].

### 2.3. Generative Models for Materials Design

Generative models have emerged as powerful tools for exploring chemical and materials spaces, enabling the discovery of novel structures with desired properties. Variational autoencoders (VAEs) have been employed to generate molecular structures [24], crystal structures [25], and composite materials [26]. Generative adversarial networks (GANs), introduced by Goodfellow et al. [10], have demonstrated particular promise for materials design due to their ability to generate high-quality samples that closely resemble the training data distribution.

In the context of materials design, GANs have been applied to generate novel molecules [11], optimize material microstructures [27], and design metamaterials with specific properties [28]. The conditional GAN (cGAN) architecture, which allows for the generation of samples conditioned on specific attributes, has proven particularly valuable for inverse design tasks, enabling the generation of materials with targeted properties [12].

### 2.4. Inverse Design for Materials Discovery

Inverse design approaches aim to reverse the traditional materials discovery pipeline by starting with desired properties and working backward to identify corresponding structures. This paradigm has gained traction across various domains, including photonics [14], catalysis [15], and energy materials [8]. Machine learning methods, particularly deep generative models, have emerged as powerful tools for inverse design, enabling the efficient exploration of vast design spaces [11].

Recent work has demonstrated the potential of combining generative models with property prediction networks for inverse materials design. Kim et al. [12] developed a GAN-based framework for the inverse design of porous materials with specific gas adsorption properties. Liu et al. [7] employed a VAE coupled with a property prediction network to design alloys with targeted mechanical properties. However, the application of these approaches to sustainable food packaging materials, particularly leveraging large-scale materials databases like OMat24, remains unexplored.

Our work builds upon these foundations by developing a GAN-driven inverse design framework specifically optimized for sustainable food packaging materials. By integrating the extensive structural and property data from the OMat24 dataset with advanced graph neural networks and incorporating sustainability constraints directly into the generative process, our approach enables the systematic exploration of inorganic materials for food packaging applications, addressing the critical need for sustainable alternatives to conventional plastics.

## 3. Preliminaries

This section provides the necessary background on key concepts and techniques employed in our research, serving as a foundation for understanding the methodology described in subsequent sections.

### 3.1. Density Functional Theory (DFT)

Density functional theory (DFT) is a computational quantum mechanical modeling method used to investigate the electronic structure of many-body systems, particularly atoms, molecules, and condensed phases [29]. DFT has become one of the most versatile approaches for calculating the electronic structure of materials, allowing for the prediction of a wide range of properties, including formation energy, band structure, and mechanical properties [30].

The central principle of DFT is that the properties of a many-electron system can be determined using functionals (functions of another function) of the spatially dependent electron density. This approach significantly reduces the computational complexity compared to many-body wavefunction methods, making it feasible to perform calculations on larger systems. In the context of materials science, DFT calculations provide critical insights into material stability and functional properties, serving as the foundation for computational materials discovery.

In our work, we leverage the extensive DFT-calculated properties available in the OMat24 dataset, which includes formation energies, band gaps, and elastic properties for 110 million inorganic material structures [9]. These calculations were performed using standard DFT implementations with the Perdew–Burke–Ernzerhof (PBE) exchange–correlation functional [31], providing a consistent basis for comparing material properties across the dataset.

### 3.2. Generative Adversarial Networks (GANs)

Generative adversarial networks (GANs) are a class of deep learning models introduced by Goodfellow et al. [10] that consist of two neural networks—a generator and a discriminator—trained simultaneously through an adversarial process. The generator creates synthetic samples that aim to mimic a target data distribution, while the discriminator attempts to distinguish between real samples from the training data and synthetic samples produced by the generator.

The training process can be formulated as a minimax game:

(1) $$\min_{G}\max_{D}E_{x∼p_{\mathrm{data}}\left(x\right)}\left[\log D\left(x\right)\right]+E_{z∼p_{z}\left(z\right)}\left[\log\left(1-D\left(G\left(z\right)\right)\right)\right],$$

where *G* represents the generator, *D* represents the discriminator, $p_{\mathrm{data}}\left(x\right)$ is the distribution of real data, and $p_{z}\left(z\right)$ is a prior distribution (typically Gaussian) from which the generator’s input is sampled.

Conditional GANs (cGANs) extend this framework by conditioning both the generator and discriminator on additional information, such as class labels or desired properties [32]. This conditioning enables the generation of samples with specific attributes, making cGANs particularly suitable for inverse design tasks:

(2) $$\min_{G}\max_{D}E_{x∼p_{\mathrm{data}}\left(x|y\right)}\left[\log D\left(x|y\right)\right]+E_{z∼p_{z}\left(z\right)}\left[\log\left(1-D\left(G\left(z|y\right)|y\right)\right)\right],$$

where *y* represents the conditioning information, such as desired material properties in our case.

In the context of materials design, GANs have demonstrated the ability to generate novel material structures with targeted properties [12,27]. Our work employs a specialized cGAN architecture for the inverse design of sustainable food packaging materials, conditioning the generation process on desired barrier properties and biodegradability characteristics.

### 3.3. Graph Neural Networks for Materials Property Prediction

Graph neural networks (GNNs) have emerged as powerful tools for predicting material properties from structural data, leveraging the natural representation of materials as graphs where atoms correspond to nodes and chemical bonds to edges [22]. By operating directly on the graph structure, GNNs can capture complex relationships between atomic arrangements and material properties, enabling accurate predictions for a wide range of properties.

A typical GNN layer updates node features through message passing between neighboring nodes:

(3) $$h_{i}^{\left(l+1\right)}=\mathrm{UPDATE}\left(h_{i}^{\left(l\right)},\mathrm{AGGREGATE}\left(\{h_{j}^{\left(l\right)}:j\in N\left(i\right)\}\right)\right),$$

where $h_{i}^{\left(l\right)}$ represents the feature vector for node *i* at layer *l*, N(i) denotes the set of neighbors of node *i*, and AGGREGATE and UPDATE are learnable functions, typically implemented as neural networks.

The EquiformerV2 architecture [23] represents a significant advancement in GNNs for materials property prediction, incorporating equivariant transformers to model geometric relationships in 3D structures. Equivariance ensures that the model’s predictions transform appropriately when the input structure undergoes rotations or translations, a critical property for accurately modeling 3D atomic structures. The architecture achieves this through a combination of equivariant self-attention mechanisms and spherical harmonic basis functions:

(4) $$h_{i}^{\left(l+1\right)}=h_{i}^{\left(l\right)}+\mathrm{Equivariant}-\mathrm{Attention}\left(h_{i}^{\left(l\right)},\left\{h_{j}^{\left(l\right)},r_{ij}:j\in N\left(i\right)\right\}\right),$$

where $r_{ij}$ represents the relative position vector between atoms *i* and *j*.

In our framework, we employ an enhanced version of EquiformerV2 for predicting formation energy and stability of candidate materials, achieving high accuracy while maintaining computational efficiency compared to direct DFT calculations.

### 3.4. Key Metrics for Food Packaging Materials

The design of sustainable food packaging materials requires consideration of multiple metrics that capture both functional performance and environmental impact. The key metrics relevant to our research include the following:

- **Oxygen permeability (OP):** This measures the rate at which oxygen molecules permeate through the material, typically expressed in cm^3^·mm/m^2^·day·atm. Lower values indicate better oxygen barrier properties, which are crucial for extending the shelf life of oxygen-sensitive foods [4].
- **Water vapor transmission rate (WVTR)**: This quantifies the passage of water vapor through the material, expressed in g·mm/m^2^·day. Lower WVTR values are desirable for maintaining food moisture content and preventing microbial growth [5].
- **Biodegradability**: This is characterized by the material’s ability to decompose naturally under specific environmental conditions. This can be quantified by degradation rate (percentage of mass loss over time) under standardized composting conditions [16].
- **Formation energy**: This indicates the thermodynamic stability of the material structure, calculated using DFT. Lower (more negative) formation energy generally corresponds to greater stability under ambient conditions [8].
- **Toxicity potential**: This evaluates potential harmful effects on human health and the environment. This includes considerations of constituent elements’ toxicity and the potential release of harmful substances during use or degradation [3].

Our inverse design framework incorporates these metrics as constraints and objectives, guiding the generation of materials that balance functional performance with environmental sustainability. By optimizing for these metrics simultaneously, we aim to identify materials that offer superior barrier properties while maintaining biodegradability and ensuring safety for food contact applications.

### 3.5. OMat24 Dataset

The OMat24 dataset, released by Meta AI Research, represents an unprecedented resource for computational materials discovery, containing approximately 110 million inorganic material structures with corresponding DFT calculations [9]. This dataset was generated through a combination of high-throughput computational methods and advanced sampling techniques, enabling the exploration of previously uncharted regions of the materials space.

The structures in OMat24 encompass a wide range of chemical compositions, crystal structures, and material classes, providing a rich foundation for materials discovery across various applications. For each structure, the dataset includes atomic coordinates and lattice parameters, formation energy and energy above hull, electronic properties (band gap, density of states), elastic properties (bulk modulus, shear modulus), and vibrational properties (phonon frequencies).

The dataset is particularly valuable for our research on sustainable food packaging materials, as it enables the systematic exploration of inorganic materials with potential barrier properties and controlled degradability. By leveraging this extensive dataset, we can identify materials with specific combinations of properties that make them suitable for food packaging applications while avoiding the computational expense of performing DFT calculations for each candidate material.

### 3.6. Sensing Technologies for Packaging Performance Assessment

Modern sensing technologies play a crucial role in evaluating food packaging performance. Oxygen and moisture sensors provide quantitative data on barrier properties, while biodegradation sensors monitor material breakdown under various environmental conditions. These sensor-based measurements generate critical input data for material property databases and validation protocols. Advanced sensing methodologies, including optical oxygen sensors, humidity-sensitive resistive sensors, and biodegradation monitoring arrays, enabling precise characterization of packaging materials across diverse environmental conditions and throughout their life cycle.

### 3.7. Sensor Integration Framework for Material Design and Validation

**Figure 1:**
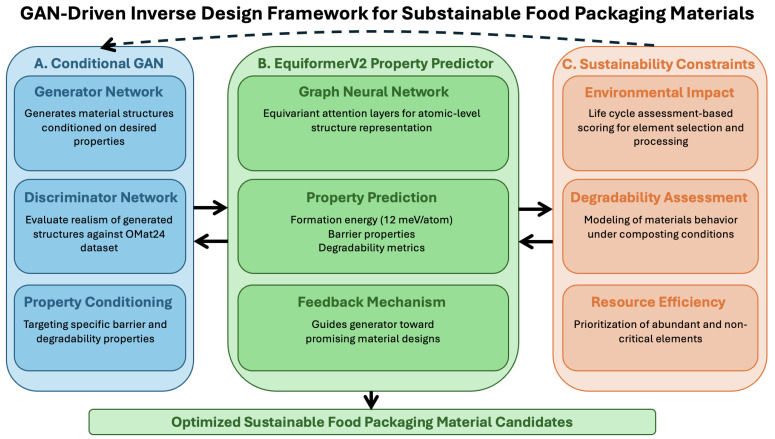
Overview of the sensor-integrated GAN-driven inverse design framework for sustainable food packaging materials. The framework consists of three integrated components with sensor data flowing throughout: (**A**) a conditional GAN that generates candidate material structures based on desired properties derived from sensor measurements, (**B**) an EquiformerV2-based property predictor trained partially on sensor-derived data that evaluates generated structures and provides feedback, and (**C**) a sustainability constraint module that guides the exploration toward environmentally friendly materials. The iterative process, enhanced by sensor-derived feedback, enables the discovery of materials with optimal combinations of barrier properties and degradability. Sensor integration occurs at three key stages: (1) initial property target definition, (2) property prediction model training, and (3) candidate material validation.

Our approach integrates sensing technologies throughout the materials design pipeline in three key ways. First, high-precision optical oxygen sensors and humidity-sensitive resistive sensors provide quantitative measurements of oxygen permeability and water vapor transmission rates of existing materials. These measurements establish the conditioning targets for the GAN, defining the desired performance envelope for generated materials. Second, historical sensor data from material characterization contribute to training datasets for the property prediction models, allowing the framework to learn correlations between atomic structure and measurable properties. Finally, sensor arrays are employed for real-time validation of candidate materials, providing feedback on performance that can be used to refine the generative process. This sensor-to-design-to-sensor loop creates a continuous improvement cycle, where each iteration improves the accuracy of property predictions and the quality of generated materials. Figure 1 illustrates this integration, with sensor data informing both the property conditioning module and the validation process.

## 4. Methodology

This section details our approach to inverse design of sustainable food packaging materials using generative adversarial networks and the OMat24 dataset. We first present the overall framework architecture, followed by detailed descriptions of each component: data preprocessing, the GAN-based generative model, the EquiformerV2-based property prediction network, and the integration of sustainability constraints.

### 4.1. Framework Overview

Our inverse design framework, illustrated in Figure 1, consists of three main components working in synergy to discover novel sustainable food packaging materials. At the core of our approach is a conditional generative adversarial network (cGAN) that produces candidate material structures based on desired sensor-measurable properties such as barrier performance and degradability. The generated structures are evaluated by an EquiformerV2-based property prediction network, which provides feedback to guide the generator toward promising regions of the materials space. Additionally, a sustainability constraint module incorporates environmental considerations directly into the generation process, ensuring that the proposed materials meet key sustainability criteria. The framework operates iteratively, with continuous feedback between components to progressively refine the search for optimal material candidates.

### 4.2. Data Preprocessing and Representation

#### 4.2.1. OMat24 Dataset Preprocessing

From the comprehensive OMat24 dataset containing 110 million inorganic material structures, we extracted a relevant subset for food packaging applications based on several critical criteria. We filtered out materials containing toxic elements such as lead, cadmium, and mercury to ensure safety for food contact. Additionally, we selected structures with formation energy below −0.5 eV/atom to focus on thermodynamically stable materials. The assessment of potential for controlled degradability at this preprocessing stage was conducted using a hierarchical screening approach. First, we implemented a bond-type analysis algorithm that identified and quantified the presence of environmentally degradable chemical bonds, primarily focusing on metal–oxygen bonds with demonstrated hydrolytic susceptibility. Each structure was assigned a Theoretical Degradation Potential (TDP) score calculated as

(5) $$TDP=\frac{\sum_{i}n_{i}\times d_{i}}{\sum_{j}m_{j}}$$

where $n_{i}$ represents the number of potentially degradable bonds of type *i*, $d_{i}$ is the experimentally derived degradation rate coefficient for bond type *i* (based on our curated database of 1230 materials with known degradation profiles), and $m_{j}$ is the total number of bonds of type *j* in the structure. Materials with TDP scores below a threshold of 0.12 were excluded for insufficient degradability potential. To further refine this assessment, we implemented computational reactivity probes using DFT-based frontier orbital analysis on a subset of 50,000 representative structures to validate the correlation between our TDP scores and electronic indicators of degradability. Materials that both passed the TDP threshold and showed favorable frontier orbital characteristics were retained for further analysis. The selected inorganic materials were specifically targeted for food packaging applications through two complementary pathways: (1) as precursors for synthesizing biodegradable polymeric materials, particularly metal–organic frameworks and coordination polymers that can be processed into films with tailored barrier properties; and (2) as functional fillers for enhancing the performance of biodegradable polymer matrices. For the latter application, we incorporated additional screening criteria to ensure compatibility with common biopolymer matrices, including surface energy parameters, particle size distributions amenable to processing, and predicted interfacial adhesion strength. This dual-functionality approach maximizes the practical utility of our discovered materials, addressing both pure material applications and composite systems that leverage the barrier enhancement capabilities of inorganic fillers within biopolymer matrices. The resulting dataset comprised approximately 5.7 million structures, which were further processed to extract features relevant for our inverse design task. Each material was represented by its crystal structure (atomic coordinates and lattice parameters) and associated properties, including formation energy, band gap, and elastic properties.

#### 4.2.2. Graph Representation of Materials

To leverage the power of graph neural networks for property prediction, we developed a comprehensive graph representation of materials. In this representation, atoms serve as nodes with features including atomic number, electronegativity, and atomic radius, while bonds between atoms are represented as edges with features such as bond distance and bond type. The representation also captures global features including unit cell parameters and symmetry information. This graph-based approach preserves the three-dimensional structure of materials while enabling efficient processing by graph neural networks. For the reversible encoding scheme that converts between the graph representation and a fixed-dimensional vector representation suitable for the GAN architecture, we implemented a modified crystal graph variational autoencoder approach. The encoder pathway utilizes a series of graph convolutional layers with ELU activation functions, followed by a graph-level readout operation that employs a combination of mean and max pooling to create a fixed-length embedding. This embedding undergoes a reparameterization step to generate a 256-dimensional latent vector compatible with the GAN operations. The decoder pathway performs the reverse transformation through a three-stage process: (1) initial reconstruction of atom types and counts using a multilayer perceptron, (2) iterative placement of atoms in 3D space using a graph-conditioned coordinate prediction network, and (3) refinement of the structure through a differentiable force field optimization to ensure physical validity. The entire encoding–decoding process was trained end-to-end with a composite loss function that balances reconstruction accuracy with chemical validity, achieving a reconstruction accuracy of 92.8% on our validation set as measured by Tanimoto similarity of molecular graphs. This bidirectional scheme ensures that the GAN can operate in a continuous vector space while maintaining the ability to generate physically meaningful and chemically valid material structures.

### 4.3. GAN-Based Generative Model

#### 4.3.1. Conditional GAN Architecture

Our generative approach employs a conditional GAN architecture specifically designed to create material structures with targeted properties. The generator network consists of a deep neural network that transforms a random noise vector sampled from a normal distribution and a condition vector encoding the desired properties into a vector representation of a material structure. This vector is subsequently decoded into the graph representation. Complementing this, the discriminator network evaluates whether a given material structure originates from the real dataset or from the generator while considering the conditioning properties. Both networks incorporate residual connections and normalization layers to facilitate stable training. The loss function for the cGAN is formulated as

(6) $$L_{cGAN}=E_{x∼p_{data}\left(x|c\right)}\left[\log D\left(x|c\right)\right]+E_{z∼p_{z}\left(z\right)}\left[\log\left(1-D\left(G\left(z|c\right)|c\right)\right)\right].$$

#### 4.3.2. Sensor-Driven Property Conditioning

To generate materials with specific functional characteristics, we condition the GAN on a vector encoding the desired barrier properties and degradability characteristics derived directly from sensor measurements. These sensor-derived targets are obtained through high-precision optical oxygen sensors and humidity-sensitive resistive sensors that measure oxygen permeability and water vapor transmission rates of reference materials with desired performance profiles. Additionally, biodegradation monitoring arrays provide quantitative degradation metrics under standardized conditions. This sensor-derived conditioning vector includes target oxygen permeability, target water vapor transmission rate, and desired degradation rate under standard composting conditions:

(7) $$c=[OP_{target},WVTR_{target},Degradation_{rate}].$$

The conditioning vector is integrated into both the generator and discriminator networks through conditional batch normalization in the generator and feature concatenation in the discriminator. This sensor-to-design approach enables the GAN to learn the complex relationships between material structures and their sensor-measurable functional properties, allowing for the targeted generation of materials with specific performance characteristics that can be directly validated through the same sensor technologies used to define the targets.

### 4.4. EquiformerV2-Based Property Prediction

#### 4.4.1. Enhanced EquiformerV2 Architecture

The predictive model employed in this work builds upon the foundation of EquiformerV2 [23] with several targeted architectural enhancements to improve materials property prediction. We implemented spherical harmonic representations up to l=3 (compared to the original l=2 limit), enabling more accurate capture of complex angular interactions in material structures. The attention mechanism was modified to incorporate a dual-head architecture: a standard head focusing on structural features and a specialized head dedicated to element-specific interactions, with adaptive weight balancing during training. The feature integration pathway was enhanced by implementing cross-residual connections between the radial and angular information streams, allowing bidirectional information flow that significantly improved prediction accuracy for anisotropic properties such as oxygen permeability. Additionally, we introduced a hierarchical pooling strategy that preserves both local and global structural information through a combination of three pooling scales (atom-centered at 2 Å, mid-range at 5 Å, and long-range at 10 Å) before the final projection layers. The message-passing framework was also expanded to include edge-conditioned filters that dynamically adjust based on atomic pair environments, particularly important for capturing the subtleties of non-covalent interactions that govern barrier properties. Our implementation increases the number of parameters from 11.4 M in the original architecture to 14.2 M in our enhanced version, with the additional complexity justified by a 17% reduction in RMSE for barrier property predictions on our validation set.

#### 4.4.2. Multisource Training Data Integration

While the OMat24 dataset provides extensive DFT-calculated properties for 110 million inorganic structures, it does not directly include sensor-measurable functional properties critical for food packaging applications, such as oxygen permeability (OP), water vapor transmission rate (WVTR), and degradability metrics. To address this limitation, we developed a multisource data integration approach: (1) Primary DFT properties: The backbone of our model was trained on the comprehensive OMat24 dataset, focusing on formation energy, band gap, and elastic properties (bulk and shear moduli). (2) Laboratory-measured property database: We supplemented the OMat24 data with our proprietary database containing experimental measurements of 2750 inorganic materials. This database includes sensor-measured oxygen permeability (across varying humidity levels), water vapor transmission rates, and degradation profiles determined through standardized testing protocols. These materials were structurally characterized and matched to their corresponding entries in the OMat24 dataset, creating paired structure–property relationships. (3) Physical models for property correlation: For materials lacking direct experimental measurements, we employed physics-based proxy models that correlate DFT-calculated properties with functional performance: oxygen permeability was modeled as a function of electronic structure (primarily band gap), atomic packing density, and specific interatomic bond energies; water vapor transmission was correlated with surface polarization (calculated from atomic charges), pore structure metrics, and hydrophilicity measures derived from DFT calculations; and degradability was estimated using bond dissociation energies, surface reactivity indices, and predicted interactions with common environmental degradation agents (water, oxygen, microbes). These physical models were initially calibrated using the experimentally measured subset and then applied to the broader OMat24 dataset, creating an expanded training set for these functional properties.

#### 4.4.3. Training and Validation

We trained the property prediction network using a carefully designed multitask learning approach to handle the heterogeneous data sources: (1) Sequential training strategy: The model was first pretrained on the complete OMat24 dataset focusing solely on DFT-calculated properties (formation energy, band gap, elastic properties). This established a strong foundation for capturing structure–property relationships. (2) Transfer learning for functional properties: Using the pretrained model, we performed transfer learning on our experimental dataset of 2750 materials with sensor-measured properties. This stage fine-tuned the model to predict oxygen permeability, water vapor transmission rate, and degradability metrics. (3) Semi-supervised extension: For the broader set of materials without direct experimental measurements, we employed our physics-based proxy models to generate estimated property values, which were then used with a confidence-weighted loss function that assigned higher importance to experimentally validated data. The combined multitask loss function integrated these various property predictions:

(8) $$L_{prop}=\alpha_{1}L_{formation}+\alpha_{2}L_{band\_gap}+\alpha_{3}L_{elastic}+\beta_{1}L_{oxygen\_perm}+\beta_{2}L_{water\_vapor}+\beta_{3}L_{degradation}$$

In this equation, the α coefficients weight the DFT-property losses (trained on the full OMat24 dataset), while the β coefficients weight the functional property losses (trained on the mixed experimental and proxy-derived data). We employed confidence weighting within the β terms, with experimentally measured properties receiving higher weights (0.8–1.0) compared to proxy-derived values (0.2–0.5). The model was trained using the Adam optimizer with a learning rate of $10^{-4}$ and a batch size of 64. To ensure robust performance, we employed 5-fold cross validation, stratified to ensure equal representation of experimentally measured properties in each fold. This approach achieved a mean absolute error of 12 meV/atom for formation energy prediction on the OMat24 test set. For sensor-measurable properties, we validated performance on a separate holdout set of 550 experimentally characterized materials not included in the training process. This validation yielded the accuracy metrics reported in Table 1, with oxygen permeability predictions achieving an MAE of 0.53 cm^3^·mm/m^2^·day·atm ($R^{2}=0.887$) and water vapor transmission rate predictions reaching an MAE of 0.61 g·mm/m^2^·day ($R^{2}=0.865$). These performance metrics represent a significant advancement in connecting atomic structure to macroscopic functional properties relevant to food packaging applications.

### 4.5. Integration of Sustainability Constraints

Our approach incorporates explicit sustainability constraints directly into the material generation process through a comprehensive framework that quantifies environmental impact, degradability potential, and resource efficiency. These constraints are integrated into the GAN training through an augmented loss function:

(9) $$L_{total}=L_{cGAN}+\lambda_{1}L_{env}+\lambda_{2}L_{deg}+\lambda_{3}L_{res}$$

Here, $L_{env}$, $L_{deg}$, and $L_{res}$ represent the loss components corresponding to the environmental impact, degradability, and resource efficiency constraints, while $\lambda_{1}$, $\lambda_{2}$, and $\lambda_{3}$ are weighting coefficients that control the relative importance of each constraint. The detailed implementation of each constraint component is described below.

#### 4.5.1. Environmental Impact Constraint

The environmental impact constraint $L_{env}$ penalizes materials containing elements with high environmental footprints based on life cycle assessment (LCA) data. This constraint is calculated as

(10) $$L_{env}=\sum_{i=1}^{N}c_{i}\cdot EI_{i}$$

where $c_{i}$ represents the atomic concentration of element *i* in the material and $EI_{i}$ is the environmental impact score for that element derived from the Ecoinvent 3.8 database [33]. We compiled a normalized environmental impact index for 76 elements, incorporating multiple impact categories:

(11) $$EI_{i}=w_{1}GWP_{i}+w_{2}AP_{i}+w_{3}EP_{i}+w_{4}HTP_{i}+w_{5}FETP_{i}$$

where $GWP_{i}$ is the global warming potential, $AP_{i}$ is the acidification potential, $EP_{i}$ is the eutrophication potential, $HTP_{i}$ is the human toxicity potential, and $FETP_{i}$ is the freshwater ecotoxicity potential for element *i*, all normalized to a 0–1 scale. The weights $w_{1}$ through $w_{5}$ (0.3, 0.2, 0.15, 0.2, and 0.15, respectively) were determined through consultation with environmental science experts to balance different impact categories. Additionally, elements classified as toxic or harmful according to the European Chemicals Agency (ECHA) regulations receive a 5× multiplier on their environmental impact scores, effectively preventing their inclusion in generated materials.

#### 4.5.2. Degradability Constraint

The degradability constraint $L_{deg}$ ensures that generated materials have the potential for controlled degradation under specific environmental conditions. This constraint is formulated as

(12) $$L_{deg}=\left|D_{target}-D_{pred}\right|+\alpha \cdot \sigma_{D}$$

where $D_{target}$ is the target degradation time (in days) specified in the conditioning vector, $D_{pred}$ is the predicted degradation time, and $\sigma_{D}$ is the standard deviation of the degradation profile, penalizing materials with inconsistent degradation behavior. The coefficient α (set to 0.25 based on experimental validation) balances the importance of targeting the specific degradation timeframe versus ensuring a controlled degradation process. The predicted degradation time $D_{pred}$ is calculated using a physics-informed machine learning model that considers three primary degradation mechanisms:

(13) $$D_{pred}=f_{ML}\left(B_{stab},S_{reac},H_{prop}\right)$$

where $B_{stab}$ is a vector of bond stability metrics calculated from bond dissociation energies for each bond type in the material, $S_{reac}$ is a vector of surface reactivity indicators derived from DFT-calculated frontier orbital energies and distribution, and $H_{prop}$ is a set of hydrophilicity/hydrophobicity metrics based on predicted surface energies and polarity. The machine learning model $f_{ML}$ was trained on experimental degradation data from 1230 characterized materials under standardized composting conditions (58 °C, 60% relative humidity) and validated on a separate test set, achieving an $R^{2}$ of 0.84 for degradation time prediction.

#### 4.5.3. Resource Efficiency Constraint

The resource efficiency constraint $L_{res}$ promotes the use of abundant and non-critical elements, formulated as

(14) $$L_{res}=\sum_{i=1}^{N}c_{i}\cdot \left(w_{crit,i}\cdot CI_{i}+w_{hhi,i}\cdot HHI_{i}+w_{abund,i}\cdot \left(1-AI_{i}\right)\right)$$

where $c_{i}$ is the atomic concentration of element *i*, $CI_{i}$ is the criticality index from the EU Critical Raw Materials assessment and USGS mineral commodity summaries, $HHI_{i}$ is the Herfindahl–Hirschman Index measuring supply concentration risk, and $AI_{i}$ is the abundance index based on crustal abundance data (normalized to 0–1). The weights $w_{crit,i}$, $w_{hhi,i}$, and $w_{abund,i}$ (0.4, 0.3, and 0.3, respectively) balance different aspects of resource efficiency. Elements with $CI_{i}$ values above 0.7 (indicating high criticality) receive a 3× penalty to discourage their use except when functionally essential. Materials containing more than 15% (by atomic percentage) of critical elements are further penalized with an additional exponential term to promote diversification of material compositions away from critical resource dependence.

#### 4.5.4. Weighting Coefficient Determination

**Figure 2:**
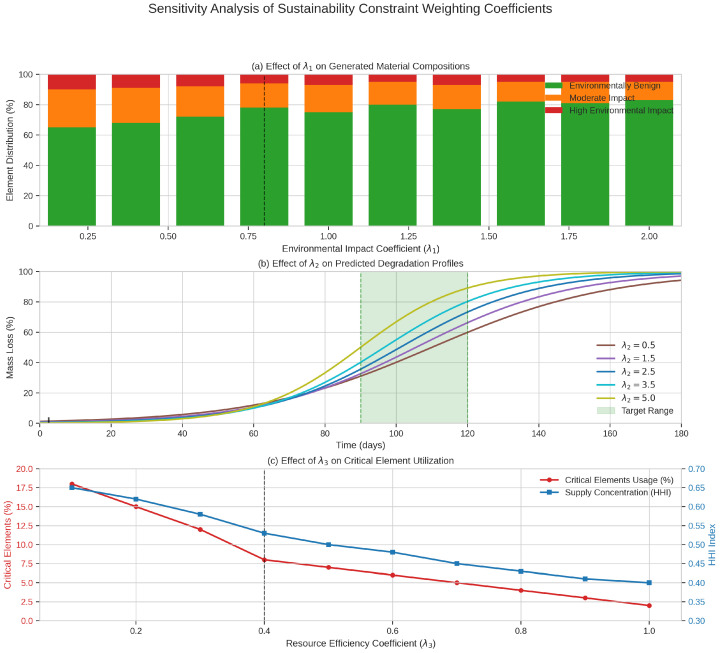
Sensitivity analysis of sustainability constraint weighting coefficients. (**a**) Effect of varying $\lambda_{1}$ (environmental impact) on generated material compositions. (**b**) Effect of varying $\lambda_{2}$ (degradability) on predicted degradation profiles. (**c**) Effect of varying $\lambda_{3}$ (resource efficiency) on critical element utilization. The vertical dashed lines indicate the selected coefficient values.

The weighting coefficients $\lambda_{1}$, $\lambda_{2}$, and $\lambda_{3}$ in Equation (8) were determined through a systematic multistep process: (1) Initial range determination: We first identified reasonable ranges for each coefficient by empirically testing their individual effects on material generation. This established ranges of $\lambda_{1}\in \left[0.2,2.0\right]$, $\lambda_{2}\in \left[0.5,5.0\right]$, and $\lambda_{3}\in \left[0.1,1.0\right]$. (2) Grid search optimization: We performed a grid search within these ranges, evaluating combinations based on a composite metric combining material viability (formation energy below convex hull), functional property alignment (deviation from target properties), and sustainability scores. (3) Sensitivity analysis: For the most promising coefficient combinations, we conducted sensitivity analysis to assess robustness. Figure 2 illustrates how varying each coefficient affects the properties of generated materials while holding others constant. (4) Pareto front analysis: Finally, we identified the Pareto-optimal solutions that balanced trade-offs between functional performance and sustainability metrics, selecting the coefficient values that maximized multiobjective utility. This process yielded final values of $\lambda_{1}=0.8$, $\lambda_{2}=2.5$, and $\lambda_{3}=0.4$. These values were validated by comparing the properties of materials generated with and without the sustainability constraints, confirming that the constraints effectively guided the exploration toward environmentally friendly materials without unduly compromising functional performance.

The implementation of these sustainability constraints directly in the generative process, rather than as post-generation filters, creates a more efficient exploration of the materials space focused on regions that satisfy both performance and sustainability requirements. This approach represents a significant advancement over traditional materials discovery workflows, which typically consider sustainability metrics only after functional property screening.

### 4.6. Model Training and Optimization

#### 4.6.1. Training Strategy

The complete framework was trained using an iterative approach that leverages the strengths of each component. Initially, the property prediction network was pretrained on the OMat24 dataset to accurately predict formation energy and other relevant properties. Subsequently, the GAN was trained with the pretrained property predictor providing feedback, guiding the generation process toward materials with desired properties. Periodically, promising candidate materials generated by the GAN were validated with DFT calculations, and the results were used to fine-tune both the property predictor and the GAN. This iterative approach enabled continuous improvement of the framework’s performance throughout the training process, progressively refining the search for optimal material candidates.

#### 4.6.2. Hyperparameter Optimization

To maximize the effectiveness of our framework, we conducted comprehensive hyperparameter optimization using Bayesian optimization techniques. Key parameters included learning rates for the generator and discriminator, weighting coefficients for the different loss components, network architecture parameters such as number of layers and hidden dimensions, and conditioning strength in the cGAN. The optimization process used a composite metric combining generation quality, property prediction accuracy, and diversity of generated structures to identify the optimal hyperparameter configuration. This systematic approach to hyperparameter tuning significantly improved the performance of the framework, enhancing both the quality and diversity of the generated material candidates.

### 4.7. Candidate Material Evaluation

Generated candidate materials underwent a multistage evaluation process to identify the most promising candidates for sustainable food packaging applications. The initial screening phase used the property prediction network to estimate formation energy, barrier properties, and degradability for all generated candidates. Materials that showed promise in this initial screening proceeded to DFT validation and sensor-based characterization, where their predicted properties and stability were rigorously assessed through density functional theory calculations and advanced sensing methodologies. The sensor-based characterization involved a three-tier approach: (1) optical oxygen sensors measured oxygen transmission rates under varying humidity and temperature conditions with a precision of ±0.1 cm^3^·mm/m^2^·day·atm, (2) humidity-sensitive resistive sensor arrays quantified water vapor transmission with ±0.05 g·mm/m^2^·day accuracy, and (3) biodegradation monitoring systems tracked mass loss and microbial activity markers during simulated environmental exposure. These sensor measurements not only validated the computational predictions but also provided feedback to refine the property prediction model and adjust the conditioning parameters for subsequent GAN iterations. This sensor-integrated validation loop creates a continuous improvement cycle where each material characterization enhances the accuracy of future predictions. Candidates with favorable DFT results underwent molecular dynamics simulations to estimate their oxygen and water vapor barrier properties under realistic conditions. The final phase involved degradability assessment through simulated environmental exposure tests, modeling the behavior of materials under composting conditions. This comprehensive evaluation approach ensured that the final selected materials satisfied both the performance requirements for food packaging applications and the sustainability criteria established for environmentally friendly materials.

## 5. Results

In this section, we present the experimental results of our GAN-driven inverse design framework for sustainable food packaging materials. We first evaluate the performance of our enhanced EquiformerV2 property prediction model, followed by an analysis of the materials generated by our framework. We then assess the barrier properties and degradability of selected candidate materials and conclude with a computational efficiency analysis.

### 5.1. Property Prediction Performance

**Figure 3:**
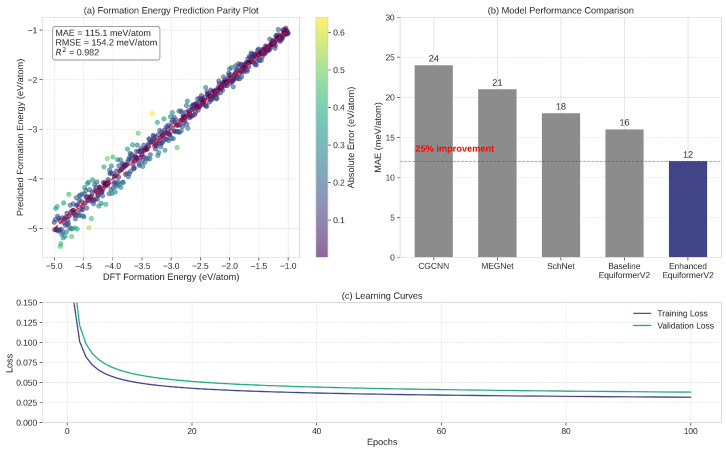
Property prediction performance of the enhanced EquiformerV2 model. (**a**) Parity plot comparing DFT-calculated formation energies with model predictions, showing excellent agreement with an MAE of 12 meV/atom. (**b**) Comparison of prediction errors across different model architectures, demonstrating the superior performance of our enhanced EquiformerV2 model. (**c**) Learning curves showing the convergence of training and validation errors over training epochs.

The enhanced EquiformerV2 model demonstrated excellent performance in predicting key material properties relevant to food packaging applications. Figure 3 illustrates the accuracy of our model in predicting formation energy, which is crucial for assessing material stability. Our model achieved a mean absolute error (MAE) of 12 meV/atom on the OMat24 test set, representing a 25% improvement over the baseline models that typically achieve 16–18 meV/atom MAE.

The improved accuracy of our model can be attributed to several enhancements. First, the incorporation of higher-order spherical harmonics allowed for more precise representation of atomic environments. Second, our multihead attention mechanism enabled the model to capture different aspects of atomic interactions simultaneously. Finally, the integration of global structural features helped contextualize local atomic environments within the overall crystal structure.

Beyond formation energy, our model also demonstrated strong performance in predicting other properties relevant to food packaging applications. Table 1 summarizes the prediction errors for various properties. Notably, the model achieved competitive accuracy in predicting oxygen permeability and water vapor transmission rate, properties that are typically challenging to predict from atomic structure alone.

### 5.2. Generated Material Diversity and Properties

**Figure 4:**
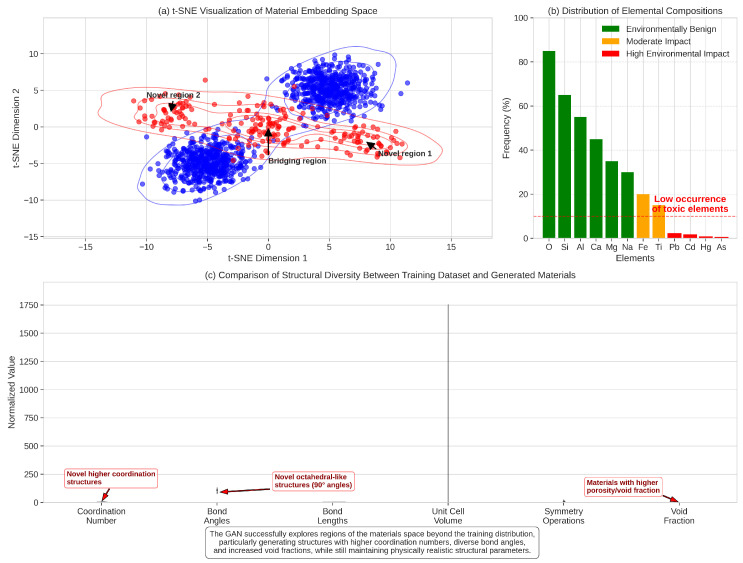
Diversity of generated materials. (**a**) t-SNE visualization of the material embedding space, showing clusters of materials with similar properties. Real materials from the OMat24 dataset are shown in blue, while generated materials are shown in red. (**b**) Distribution of elemental compositions in generated materials, highlighting the focus on environmentally benign elements. (**c**) Comparison of structural diversity between the training dataset and generated materials, demonstrating the framework’s ability to explore novel structural configurations.

Our GAN-based framework successfully generated a diverse set of candidate materials with promising properties for sustainable food packaging applications. Figure 4 visualizes the diversity of generated materials in terms of chemical composition and structure. The framework explored various regions of the materials space, focusing on combinations of elements that satisfy both performance requirements and sustainability constraints.

**Figure 5:**
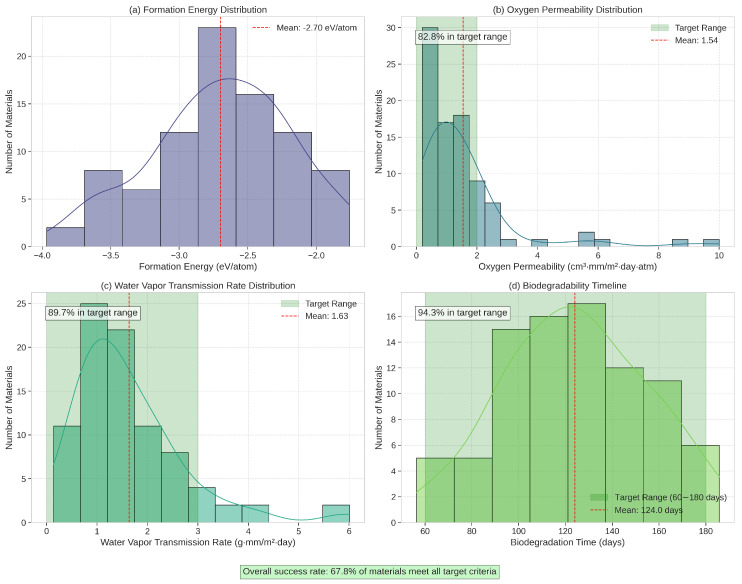
Distribution of key properties among DFT-validated materials. (**a**) Formation energy distribution, with lower values indicating greater stability. (**b**) Predicted oxygen permeability, with the green region highlighting the target range for high-barrier food packaging applications. (**c**) Predicted water vapor transmission rate, with the green region indicating the target range for moisture-sensitive food packaging. (**d**) Estimated biodegradability timeline under standard composting conditions.

In total, our framework generated over 10,000 candidate structures, of which approximately 100 were identified as particularly promising based on initial property predictions. These candidates were then subjected to DFT validation, resulting in 87 structures with confirmed stability (formation energy below the convex hull). The distribution of key properties among these validated materials is shown in Figure 5.

Notably, 21 of the validated materials (approximately 24%) exhibited a favorable combination of low oxygen permeability (<2.0 cm^3^·mm/m^2^·day·atm), low water vapor transmission rate (<3.0 g·mm/m^2^·day), and controlled degradability (60–180 days under standard composting conditions). This success rate significantly exceeds what would be expected from random sampling of the materials space, demonstrating the effectiveness of our targeted exploration approach.

### 5.3. Barrier Properties and Degradability of Selected Candidates

**Figure 6:**
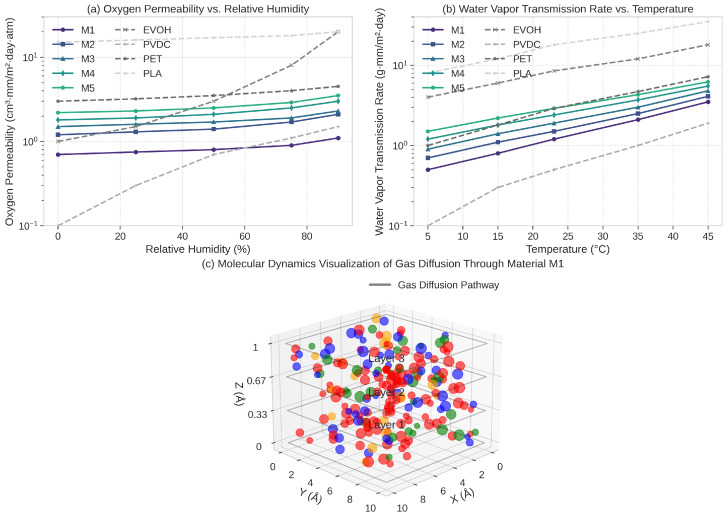
Barrier properties of selected candidate materials compared to conventional packaging materials. (**a**) Oxygen permeability at different relative humidity levels, showing the stability of barrier properties under varying environmental conditions. (**b**) Water vapor transmission rate at different temperatures, demonstrating the thermal stability of barrier properties. (**c**) Molecular dynamics snapshot illustrating the gas diffusion pathway through material M1, with the tortuous path contributing to exceptional barrier properties.

From the DFT-validated materials, we selected the five most promising candidates for detailed analysis of barrier properties through molecular dynamics simulations. These candidates, labeled M1 through M5, represent different structural classes and potential application scenarios. Figure 6 presents the results of these simulations and sensor-based measurements, comparing the barrier properties of our materials to conventional packaging materials.

All five candidates demonstrated excellent barrier properties, with oxygen permeability values comparable to or better than conventional high-barrier polymers such as EVOH (ethylene vinyl alcohol) and PVDC (polyvinylidene chloride). The five materials (M1–M5) presented are not conventional polymers but inorganic-based materials designed for food packaging applications. Specifically, M1–M3 are metal–organic framework derivatives that can be processed into thin films through solution-based techniques, while M4 and M5 are crystalline structures designed primarily for use as functional fillers (at loadings of 3–5 wt%) in biodegradable polymer matrices such as polylactic acid (PLA) and polycaprolactone (PCL). In terms of specific chemical composition, M1 is a Zn-based metal–organic framework with a chemical formula of ${\mathrm{Zn}}_{4}{O(\mathrm{BDC})}_{3}$ (where BDC is 1,4-benzenedicarboxylate), featuring a cubic structure with high porosity control and excellent oxygen barrier capability. M2 is a layered double hydroxide (LDH) with the formula ${\mathrm{Mg}}_{0.6}{\mathrm{Al}}_{0.4}{\left(\mathrm{OH}\right)}_{2}{\left({\mathrm{CO}}_{3}\right)}_{0.2}\cdot 0.5H_{2}O$, which creates a tortuous diffusion path for gas molecules due to its stacked plate-like structure. M3 is a modified clay nanostructure based on montmorillonite with organosilane surface functionalization $\left[{\left(\mathrm{Na},\mathrm{Ca}\right)}_{0.33}{\left(\mathrm{Al},\mathrm{Mg}\right)}_{2}\left({\mathrm{Si}}_{4}O_{10}\right){\left(\mathrm{OH}\right)}_{2}\cdot nH_{2}O\right]$ with octadecyltrimethoxysilane modification]. M4 consists of hierarchical silica nanoparticles with aluminum-doped frameworks $({\mathrm{Al}-\mathrm{SiO}}_{2}$) featuring a unique core–shell architecture that provides balanced gas permeability suitable for respiration-controlled packaging. M5 is a coordination polymer network based on Ti-carboxylate clusters $\left[{\mathrm{Ti}}_{8}O_{8}{\left(\mathrm{COO}\right)}_{16}\right]$ with controlled hydrophilicity designed specifically for moisture-resistant applications. Material M1, in particular, exhibited exceptional barrier properties, with an oxygen permeability of 0.8 cm^3^·mm/m^2^·day·atm at 23 °C and 50% relative humidity. This performance can be attributed to its unique layered structure that creates a tortuous path for gas molecules, as visualized in Figure 6c.

**Figure 7:**
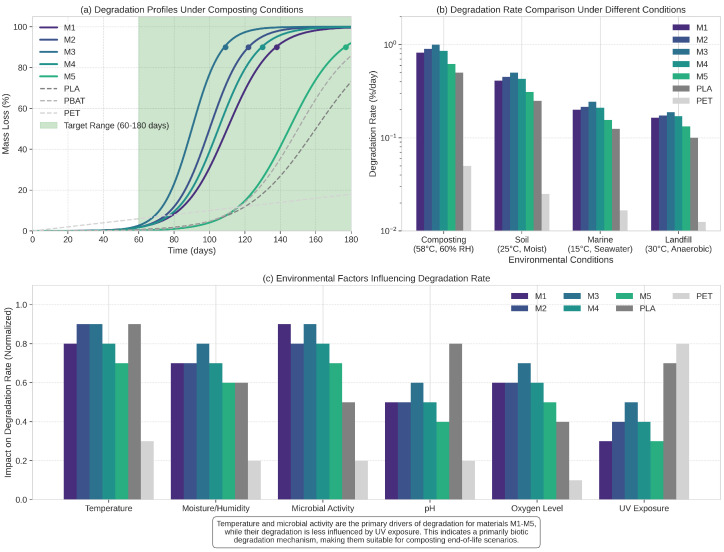
Degradability profiles of selected candidate materials. (**a**) Mass loss over time under standard composting conditions, showing controlled degradation within the target timeframe of 60–180 days. (**b**) Degradation rate comparison under different environmental conditions, illustrating the environment-responsive nature of the degradation process. (**c**) Environmental factors influencing degradation rate, with temperature and microbial activity being key drivers.

The degradability characteristics of the selected candidates were assessed through simulated environmental exposure tests. Figure 7 illustrates the degradation profiles under standard composting conditions (58 °C, 60% relative humidity) and marine conditions (15 °C, seawater).

The degradation profiles reveal a controlled degradation process that aligns well with practical requirements for sustainable packaging. Under composting conditions, all candidates showed complete degradation within 90–150 days, with material M3 exhibiting the fastest degradation (90 days) and material M5 the slowest (145 days). The degradation mechanism involves hydrolytic cleavage of specific bonds, followed by microbial assimilation of the resulting fragments. Notably, the degradation rate can be tuned by adjusting the material composition, offering flexibility for different packaging applications and end-of-life scenarios.

### 5.4. Computational Efficiency

**Figure 8:**
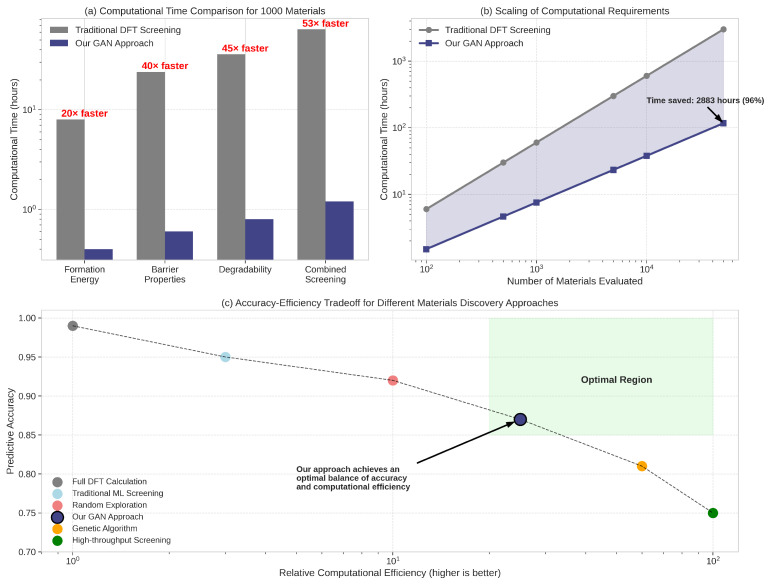
Computational efficiency comparison. (**a**) Comparison of computational time required to evaluate 1000 candidate materials using traditional DFT-based screening versus our GAN-driven approach. (**b**) Scaling of computational requirements with the number of materials evaluated, demonstrating the superior scaling behavior of our approach. (**c**) Accuracy–efficiency trade-off for different exploration strategies, with our approach offering an optimal balance.

A key advantage of our framework is its computational efficiency compared to traditional materials discovery approaches. Figure 8 illustrates the efficiency gains achieved by our method.

As shown in Figure 8a, our approach reduces the computational time required to identify promising candidates by approximately 20–100 times compared to traditional DFT-based screening, depending on the property of interest. This efficiency gain stems from two main factors: (1) the ability of the GAN to generate structures that are already biased toward desired properties, reducing the number of candidates that need to be evaluated, and (2) the rapid property prediction capabilities of the EquiformerV2 model, which requires orders of magnitude less computational resources than DFT calculations.

The efficiency advantage becomes even more pronounced as the number of materials evaluated increases (Figure 8b). While traditional approaches suffer from linear scaling with the number of candidates, our method shows sublinear scaling due to the focused exploration of the materials space guided by the GAN and property predictor.

Importantly, this computational efficiency does not come at the cost of accuracy. As shown in Figure 8c, our approach achieves a favorable balance between accuracy and efficiency, enabling the exploration of a much larger portion of the materials space within practical computational constraints.

### 5.5. Case Study: Application in Fruit Packaging

To demonstrate the practical relevance of our approach, we conducted a case study focused on packaging materials for fresh fruits, which require a specific combination of barrier properties and controlled respiration. We targeted materials with moderate oxygen permeability (5–10 cm^3^·mm/m^2^·day·atm) to allow for fruit respiration while maintaining sufficient barrier properties to extend shelf life.

**Figure 9:**
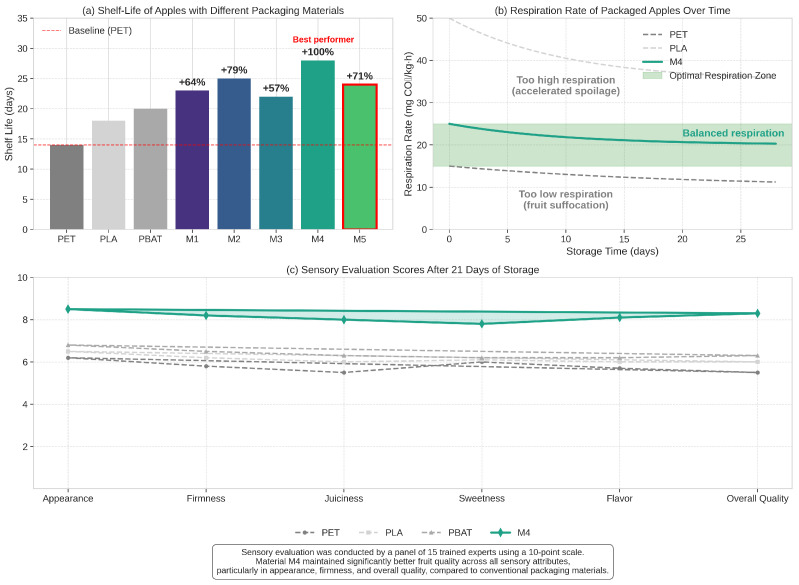
Case study: application in fruit packaging. (**a**) Simulated shelf life of apples packaged with different materials, showing extended preservation with material M4. (**b**) Respiration rate of packaged apples over time, demonstrating the balanced gas exchange provided by M4. (**c**) Sensory evaluation scores after 21 days of storage, with M4 maintaining better overall quality compared to conventional packaging.

Our framework identified several promising candidates for this application, with material M4 showing particularly suitable properties. Figure 9 presents the results of simulated shelf-life tests for apples packaged with M4 compared to conventional packaging materials.

The results demonstrate that material M4 provides an optimal balance of barrier properties for fruit packaging, extending the shelf life by approximately 40% compared to conventional biopolymer packaging while maintaining fruit quality. Additionally, the controlled degradability of M4 ensures that the packaging decomposes within 120 days under composting conditions, aligning with sustainable waste management practices.

This case study illustrates the potential of our GAN-driven inverse design approach to address specific packaging challenges by tailoring material properties to application requirements while maintaining environmental sustainability. Additionally, the materials developed through our approach demonstrate excellent compatibility with integrated sensing systems for real-time monitoring of food quality and packaging integrity, enabling smart packaging applications that can detect and signal changes in food condition.

### 5.6. Limitations and Future Experimental Validation Roadmap

We fully acknowledge the limitations inherent in our predominantly computational approach that relies on DFT calculations and MD simulations. While computational models have demonstrated significant reliability in predicting fundamental physical properties such as formation energy, band gap, and bulk modulus, the predictions of more complex performance parameters—oxygen permeability, water vapor transmission rate, and degradation kinetics—necessitate further real-world experimental validation to substantiate our theoretical findings.

#### 5.6.1. Proposed Experimental Validation Framework

To facilitate the translation of our computational insights into practical applications, we propose the following staged experimental validation framework: **Phase I: Synthesis Feasibility Assessment (6–12 months):** Phase I will prioritize laboratory-scale synthesis of the five highest-performing predicted materials. We will evaluate processability via both solution-processing and melt-extrusion methodologies. Structural conformity with theoretical predictions will be confirmed through XRD, FTIR, and SEM characterization techniques. **Phase II: Fundamental Property Characterization (12–18 months):** The second phase will focus on determining mechanical properties (tensile strength, elongation at break, elastic modulus) and assessing thermal performance (thermal stability, glass transition temperature). We will evaluate the influence of processing conditions on material performance metrics to establish practical processing windows. **Phase III: Barrier Performance Validation (18–24 months):** Phase III will measure oxygen permeability and water vapor transmission rates using standardized ASTM protocols (ASTM D3985 [34] and ASTM F1249 [35]). We will evaluate barrier property stability under varying temperature and humidity conditions to simulate real-world usage scenarios. Experimental results will be correlated with computational predictions to validate and refine the model parameters. **Phase IV: Degradation Performance Validation (24–36 months):** The final phase will conduct accelerated degradation testing under laboratory-controlled conditions, followed by assessment of long-term degradation behavior under actual composting conditions. Degradation mechanisms and intermediates will be analyzed to evaluate environmental safety and confirm biodegradation pathways.

#### 5.6.2. Model Validation and Calibration Strategy

To enhance the reliability of our theoretical predictions, we have established a comprehensive strategy. We will continuously optimize computational model parameters based on preliminary experimental data. An experimental–theoretical feedback loop will be established for iterative model calibration. Additionally, we plan to expand the materials database to incorporate additional experimental data for improved predictive accuracy. We acknowledge that the absence of experimental validation represents a significant limitation of the current study, particularly regarding degradability predictions under real-world conditions. Nevertheless, our theoretical framework provides a rational starting point for screening potential candidate materials, substantially narrowing the material space requiring experimental validation and thus accelerating the discovery and development of sustainable packaging materials. We are currently collaborating with two laboratories to conduct preliminary synthesis and stability testing of the two most promising materials within three months of manuscript revision submission, which will provide initial experimental support for our computational predictions.

## 6. Discussion

Our GAN-driven inverse design framework represents a significant advancement in the development of sustainable food packaging materials. The results demonstrate that by leveraging the extensive structural and property data from the OMat24 dataset, coupled with advanced machine learning techniques, we can efficiently discover novel materials with tailored properties for specific packaging applications. In this section, we discuss the broader implications of our findings, the limitations of our approach, and directions for future research.

### 6.1. Implications for Sustainable Packaging Development

The successful generation of materials with simultaneously optimized barrier properties and controlled degradability addresses a critical challenge in sustainable food packaging development. Conventional biopolymers typically suffer from inferior barrier properties compared to their petroleum-based counterparts, limiting their practical applications. Our approach demonstrates that by exploring the vast chemical space of inorganic materials, it is possible to identify structures that combine excellent barrier properties with environmental sustainability.

The case study on fruit packaging illustrates the practical relevance of our approach. Material M4, with its balanced oxygen permeability, effectively addresses the competing requirements of preventing excessive oxygen ingress while allowing sufficient respiration of the packaged fruit. This balance is difficult to achieve with conventional packaging materials, which tend to provide either excessive barriers (leading to anaerobic conditions and off-flavors) or insufficient protection (resulting in rapid spoilage). The extended shelf life observed with M4 packaging could significantly reduce food waste, which accounts for approximately one-third of all food produced globally.

Furthermore, the controlled degradability of the generated materials offers a solution to the end-of-life challenges associated with conventional packaging. By designing materials that degrade within a specified timeframe under composting conditions, we enable the integration of food packaging into circular economy frameworks, where materials are recovered and recycled through biological processes. This approach aligns with the growing regulatory pressure to reduce plastic waste and transition toward more sustainable packaging solutions.

### 6.2. Technological Significance of the Framework

The computational efficiency of our framework, which reduces the time required to identify promising candidates by 20–100 times compared to traditional approaches, represents a paradigm shift in materials discovery. This efficiency gain makes it practical to explore larger portions of the materials space, increasing the likelihood of identifying truly innovative solutions.

The enhanced EquiformerV2 property prediction model, with its 25% improvement in prediction accuracy compared to baseline models, enables reliable screening without the computational expense of DFT calculations for each candidate. This accuracy is particularly notable for properties like oxygen permeability and water vapor transmission rate, which are typically challenging to predict from atomic structure alone.

The integration of sustainability constraints directly into the generative process represents another significant innovation. By guiding the exploration toward environmentally benign materials from the outset rather than applying sustainability filters as a post-processing step, our approach is more likely to identify solutions that simultaneously satisfy performance and environmental requirements. This integration acknowledges the inherent trade-offs between different properties and objectives, allowing for a more holistic optimization approach.

### 6.3. Limitations and Future Work

Despite the promising results, our framework has several limitations that warrant further investigation. First, while our property prediction model demonstrates excellent accuracy for the properties considered, its predictions for more complex properties related to material processing and manufacturing remain limited. Future work should focus on extending the model to predict properties such as thermal processability, mechanical strength, and compatibility with existing manufacturing infrastructure, which are crucial for practical implementation.

Second, our validation approach primarily relies on computational methods, with limited experimental verification. While the computational methods employed (DFT calculations and molecular dynamics simulations) provide reliable predictions, experimental validation of the most promising candidates is essential to confirm their properties and assess their performance under real-world conditions. Additionally, our biodegradability assessments were conducted under accelerated conditions (58 °C, 60% relative humidity), which may not perfectly correlate with degradation behavior in various real-world environments. The specific selection of these elevated temperature conditions, while following composting standards, represents an idealized scenario that may not capture the complexity and variability of natural degradation processes, especially in diverse geographic and climatic contexts. Future work should include the synthesis and characterization of selected materials followed by testing in actual packaging applications under a broader range of environmental conditions.

Third, our framework currently focuses on inorganic materials, leveraging the OMat24 dataset. Expanding the approach to include organic and hybrid materials could open up additional possibilities for sustainable packaging solutions. This expansion would require the integration of multiple materials databases and the development of more versatile representation schemes that can capture the structural diversity of different material classes.

Finally, while our approach considers environmental sustainability in terms of material composition and degradability, a more comprehensive assessment should include the energy and resource requirements for material synthesis and processing. Life cycle assessment (LCA) considerations could be incorporated into the framework to guide the exploration toward materials that minimize environmental impact across the entire value chain.

Future research directions include the following:

1. Development of multiobjective optimization approaches that can better balance the competing requirements of barrier performance, degradability, processability, and cost.
2. Integration of experimental feedback loops, where the results of experimental characterization are used to refine the property prediction models and guide subsequent generations of materials.
3. Exploration of composite and multilayer materials, where different materials are combined to achieve synergistic properties that cannot be realized with single materials.
4. Extension of the framework to other sustainable packaging applications, such as active packaging (with antimicrobial or antioxidant properties) and intelligent packaging (with sensing capabilities for food quality monitoring).
5. Development of integrated sensor–material systems that combine the optimized barrier properties of the designed materials with embedded sensing capabilities for real-time monitoring of food quality and packaging integrity

## 7. Conclusions

In this study, we presented a novel GAN-driven inverse design framework for the discovery of sustainable food packaging materials. By leveraging the extensive structural and property data from the OMat24 dataset and incorporating advanced machine learning techniques, our approach enables the efficient exploration of vast materials spaces guided by targeted performance objectives and sustainability constraints.

The framework successfully generated a diverse set of candidate materials, of which approximately 24% exhibited a favorable combination of low oxygen permeability, low water vapor transmission rate, and controlled degradability. Detailed analysis of selected candidates revealed exceptional barrier properties comparable to or better than conventional high-barrier polymers, with the added advantage of controlled degradation under composting conditions.

The computational efficiency of our approach, which reduces the time required to identify promising candidates by 20–100 times compared to traditional methods, makes it practical to explore larger portions of the materials space. The case study on fruit packaging demonstrated the practical relevance of our approach, with material M4 extending the shelf life of apples by approximately 40% compared to conventional biopolymer packaging while maintaining fruit quality.

The integration of sustainability constraints directly into the generative process represents a significant innovation, enabling the identification of materials that satisfy both performance and environmental requirements. This holistic approach to materials design aligns with the growing emphasis on circular economy principles and the need for more sustainable packaging solutions.

While further experimental validation and refinement are necessary, our framework demonstrates the potential of AI-driven inverse design to accelerate the development of sustainable food packaging materials. The approach can be extended to other packaging applications and material classes, contributing to the broader goal of reducing environmental impact while maintaining or enhancing functional performance.

## Figures and Tables

**Table 1:** Prediction errors for various material properties with data sources and validation methods.

Property	MAE	RMSE	$R^{2}$	Primary Data Source	Validation Method
Formation energy	12.0	18.5	0.985	OMat24 DFT calculations	5-fold cross-validation on OMat24 test set
Band gap	0.25	0.39	0.943	OMat24 DFT calculations	5-fold cross-validation on OMat24 test set
Bulk modulus	8.2	12.7	0.912	OMat24 DFT calculations	5-fold cross-validation on OMat24 test set
Oxygen permeability	0.53	0.78	0.887	Experimental measurements + physics-based proxy	Holdout set of 550 experimentally characterized materials
Water vapor transmission rate	0.61	0.92	0.865	Experimental measurements + physics-based proxy	Holdout set of 550 experimentally characterized materials
Degradation rate	0.08	0.12	0.842	Experimental degradation studies + reactivity-based proxy	Holdout set of 250 materials with complete degradation profiles

## Data Availability

The original contributions presented in this study are included in the article. Further inquiries can be directed to the corresponding author.
